# 767. Impact of Standardized Patient Simulation on Pharmacy Students' Confidence and Perceptions in Community-Acquired Bacterial Meningitis Management

**DOI:** 10.1093/ofid/ofad500.828

**Published:** 2023-11-27

**Authors:** Wesley D Kufel, Keri Mastro, Bruce Blaine

**Affiliations:** Binghamton University School of Pharmacy Sciences, Binghamton, New York; Binghamton University School of Pharmacy and Pharmaceutical Sciences, Endwell, New York; University of Rochester, Rochester, New York

## Abstract

**Background:**

Limited data describes the use of standardized patient simulation (SPS) for infectious disease (ID) education, particularly among pharmacy students. We sought to assess the impact of SPS on pharmacy students’ knowledge and confidence of community-acquired bacterial meningitis (CABM), and to evaluate students’ perceptions of SPS.

**Methods:**

This single-center, cross-sectional, voluntary, anonymous survey was conducted among second-year Doctor of Pharmacy students (n=69). CABM education was initially delivered via didactic, large classroom instruction, which occurred 1.5 weeks prior to the SPS. The pre-SPS survey asked students to assess their confidence in CABM. The confidence questions focused on pathophysiology, microbiology, clinical presentation, diagnosis, and empiric/targeted management. The SPS included two group graded sections: a rounding experience focused on patient assessment, clinical presentation, and empiric management (Figure 1) followed by a microbiology lab simulation, targeted management, and duration/monitoring. A post-SPS survey was distributed, which included the same pre-survey questions and questions related to perceptions of SPS. All survey confidence and perception questions were evaluated using a Likert scale from strongly disagree (1) to strongly agree (5). Descriptive statistics and Student’s t-test were used.

Standardized Patient Simulation Rounding Experience

**Figure:**
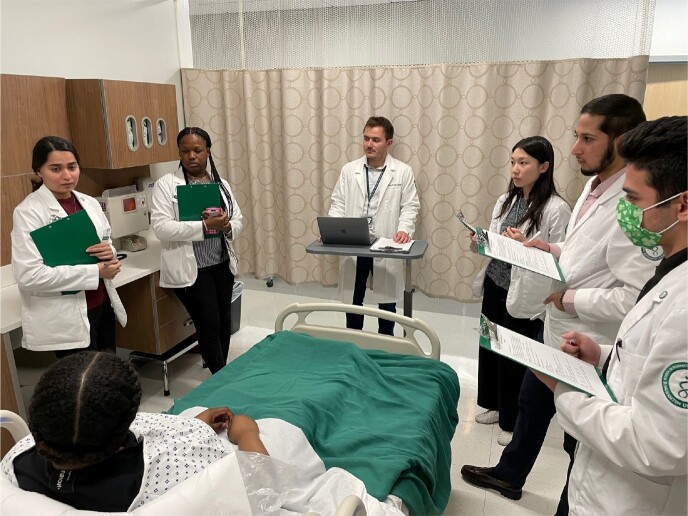

Student group engaged in the standardized patient simulation rounding experience (all individuals pictured gave permission for the use of this picture).

**Results:**

Sixty-three students fully completed the pre- and post-SPS surveys (91.3% response rate), and pre-post responses were matched. Overall mean (+SD) confidence in CABM management was significantly higher following SPS (4.3+0.5 vs. 3.5+0.7; p< 0.001). A statistically significant mean increase was also observed for each individual confidence item following SPS. Mean (+SD) perceptions following SPS for feeling more comfortable with patient interaction skills, preferring SPS compared to written case-based instruction, and having interest in more ID SPS were 4.2+0.8, 4.2+0.9, and 4.3+0.9, respectively.

**Conclusion:**

SPS was associated with increased confidence in CABM management among pharmacy students. Students appeared to have a positive impression of SPS and are interested in future ID SPS. SPS appears to be an effective educational modality for CABM and should be assessed among other student clinicians for ID.

**Disclosures:**

**Wesley D. Kufel, Pharm.D., BCPS, BCIDP, AAHIVP**, Merck & Co.: Grant/Research Support

